# Meteorological Observations, September

**Published:** 1860-10

**Authors:** J. G. Westmoreland


### METEOROLOGICAL OBSERVATIONS,

Taken by J. G. Westmoreland, M. D., for the Smithsonian Institution, September, 1860, at Atlanta, Ga., Latitude 33 deg. 45 min.—Longitude 7 deg. 30 min.—Height above the Sea, 1050 feet.

| Days of Month | Barometer. 7 A.M. | Barometer. 2 P.M. | Barometer. 9 P.M. | Thermometer. 7 A.M. | Thermometer. 2 P.M. | Thermometer. 9 P.M. | Rain and Snow. Amt. Rn | Rain and Snow. In. Sn'w | Clouds. 7 A.M. | Clouds. 2 P.M. | Clouds. 9 P.M. | Wind. 7 A.M. | Wind. 2 P.M. | Wind. 9 P.M. |
|---|---|---|---|---|---|---|---|---|---|---|---|---|---|---|
| 1 | 28.80 | 28.90 | 28.95 | 70 | 86 | 80 | | | 0 | 0 | 0 | W | W | W |
| 2 | 28.90 | 29.00 | 28.90 | 70 | 86 | 78 | 1.00 | | 0 | 5 | 10 | S W | S W | S |
| 3 | 29.00 | 29.00 | 29.00 | 70 | 70 | 72 | .60 | | 10 | 10 | 10 | S | W | E |
| 4 | 29.05 | 29.05 | 29.00 | 70 | 80 | 76 | | | 0 | 5 | 5 | S E | E | E |
| 5 | 29.00 | 29.00 | 28.95 | 70 | 78 | 70 | | | 5 | 5 | 0 | E | E | E |
| 6 | 28.95 | 28.95 | 28.95 | 70 | 80 | 76 | | | 0 | 0 | 0 | E | E | E |
| 7 | 28.95 | 29.00 | 28.95 | 70 | 82 | 74 | | | 0 | 0 | 0 | E | N E | N E |
| 8 | 28.95 | 28.95 | 28.90 | 70 | 84 | 76 | | | 0 | 0 | 0 | N E | N E | W |
| 9 | 28.85 | 28.90 | 28.85 | 72 | 88 | 78 | | | 0 | 0 | 0 | N W | N W | N W |
| 10 | 28.80 | 29.80 | 28.80 | 68 | 70 | 68 | | | 10 | 10 | 10 | E | E | E |
| 11 | 28.85 | 28.90 | 28.90 | 66 | 80 | 68 | | | 5 | 0 | 0 | E | N E | N W |
| 12 | 28.90 | 28.92 | 28.95 | 60 | 76 | 64 | | | 0 | 0 | 0 | N E | N E | N E |
| 13 | 29.00 | 29.05 | 29.00 | 58 | 72 | 60 | | | 0 | 0 | 0 | E | E | E |
| 14 | 29.05 | 29.12 | 29.10 | 54 | 76 | 70 | | | 0 | 0 | 0 | N W | N W | N W |
| 15 | 29.10 | 29.10 | 29.00 | 62 | 78 | 68 | | | 0 | 5 | 5 | N E | N E | E |
| 16 | 28.95 | 28.90 | 28.80 | 64 | 72 | 68 | .75 | | 5 | 10 | 10 | E | E | E |
| 17 | 28.70 | 28.70 | 28.75 | 66 | 68 | 62 | | | 10 | 10 | 10 | E | W | N W |
| 18 | 28.80 | 28.85 | 28.85 | 62 | 72 | 66 | | | 10 | 5 | 5 | N | W | W |
| 19 | 28.85 | 28.90 | 28.80 | 66 | 78 | 72 | | | 10 | 5 | 5 | W | W | W |
| 20 | 28.80 | 28.80 | 28.85 | 70 | 78 | 54 | | | 5 | 5 | 0 | W | W | N W |
| 21 | 28.85 | 28.95 | 28.90 | 50 | 66 | 60 | | | 0 | 0 | 0 | N W | N W | W |
| 22 | 28.95 | 29.00 | 28.95 | 50 | 70 | 64 | | | 0 | 0 | 0 | W | S W | S W |
| 23 | 28.90 | 29.00 | 28.90 | 52 | 72 | 66 | | | 0 | 0 | 0 | S | S W | S |
| 24 | 28.90 | 28.92 | 28.90 | 60 | 78 | 68 | | | 0 | 5 | 0 | S | S | W |
| 25 | 28.85 | 28.95 | 29.00 | 64 | 82 | 70 | | | 0 | 0 | 0 | W | W | W |
| 26 | 29.00 | 29.05 | 29.00 | 68 | 84 | 70 | | | 0 | 0 | 0 | W | W | W |
| 27 | 29.00 | 29.10 | 29.05 | 64 | 86 | 72 | | | 0 | 0 | 0 | W | W | W |
| 28 | 29.00 | 29.00 | 29.00 | 76 | 84 | 72 | | | 0 | 0 | 0 | W | W | W |
| 29 | 29.00 | 29.00 | 28.95 | 64 | 70 | 60 | | | 10 | 10 | 10 | E | E | E |
| 30 | 28.95 | 29.00 | 29:00 | 58 | 70 | 64 | | | 10 | 0 | 0 | E | E | E |

Explanation of Table.—0 Signifies Entire Clearness—5 Half Cloudy—10 Entire Cloudiness.

☞ The Barometer used in taking these Meteorological observations is the Aneroid instrument, and being set one and a fourth degree higher than the Mercurial Barometer has run to that extent higher than the tables made from it. The index is now set with the Mercurial Barometer commencing with this table.

